# Effect of mechanochemical activation of natural phosphorite structure as well as phosphorus solubility

**DOI:** 10.1371/journal.pone.0224423

**Published:** 2019-11-07

**Authors:** Nana Fang, Yuanliang Shi, Zhenhua Chen, Xun Sun, Lei Zhang, Yanli Yi

**Affiliations:** 1 College of Land and Environment, Shenyang Agricultural University, Shenyang, China; 2 Institute of Applied Ecology, Chinese Academy of Sciences, Shenyang, China; Institute of Soil Science, CHINA

## Abstract

Mechanochemical treatment of phosphate rock is considered as an effective and ecologically clean way of treating the medium- and low-grade phosphorite which could be used as fertilizer instead of the high-grade phosphorite. In order to investigate the effects of different milling times on the mechanochemically activated phosphorite (lower total phosphorus content) by more efficient milling equipment with enhanced milling speed, phosphorus solubility in citric acid and structural characteristics of natural and mechanochemically activated phosphorite from Yichang, China were studied using scanning electron microscope, infrared spectroscopy and X-ray diffraction. Phosphorus solubility in citric acid increased proportionately with the milling time until 30 min (57.51%), but then gradually reached an equilibrium with the maximum (59.03%) in 50 min. These changes were mainly manifested in considerably reduced particle size, decreased crystallinity and increased structural defects of phosphorite due to substitution of PO_4_^3-^ with CO_3_^2-^ and the incorporation of OH^-^. With the incorporation of CO_3_^2-^ and OH^-^, the non-activated carbonate-fluorapatite (type B) was transformed into a mixture of carbonate-fluorapatite, hydroxyapatite, fluorocarbon hydroxyapatite and/or carbonate apatite, respectively during the process of mechanochemical activation. As a result of the structural and phase transformations after mechanochemical activation, phosphorus solubility remarkably increased.

## Introduction

Currently, 70–80% of high-grade phosphorite (P_2_O_5_≥30%) in the world has been used to produce phosphorus fertilizer to ensure enough nutrition for crops. The depletion of high-grade phosphorite as well as the ecological problems caused by the wet treatment with acid have intensified the problem of finding a more efficient ways of producing phosphorus fertilizers. Medium- and low-grade phosphorite (P_2_O_5_<30%) can not be directly used as phosphate fertilizers, unless after processing, upgrading or flotation which require complex techniques, and high-cost reagents [1]. Finding a way to utilize this abandoned medium- and low-grade phosphorite has been a concern in recent years. Mechanochemical treatment is characterized by simplicity, ecological cleanliness, lack of the need for flotation or chemicals, and no waste generation which is usually done increase the reactivity of natural and synthetic apatite minerals [2]. The reasons for the increasing assimiable phosphorus from apatite by mechanochemical activation should be either structural changes or phase transformations in the treated apatite [3,4]. Mechanochemical activation influences the reactivity of apatite not only due to decreased particle size, but also due to increased structural defects because of the incorporation of CO_2_ (from the air) and formation of OH^-^ in the apatite structure [5].

Previous researches mainly focused on the optimization of the processing parameters of milling equipment [6] and on structure analysis with infrared spectroscopy (IR) and X-ray diffraction (XRD) methods [7, 8]. In our previous study, the physical and chemical characteristics of Huangmailing phosphite (total phosphorus solubility in citric acid, P_2_O_5_27.4%) after mechanical activation were compared with the initial ore by scanning electron microscope (SEM) and XRD [9]. However, little work has been done on the effectiveness and structural changes of phosphorite with lower total phosphorus content, more efficient milling equipment decreased milling time and enhanced milling speed. The mean of selecting the effective milling time needs to be discussed, considering the energy consumption in industrial production of phosphorus fertilizer in the future.

The aim of our research was to elucidate the solubility and structural changes in the process of mechanochemical activation of a typical medium- and low-grade phosphorite with 22% P_2_O_5_ from Yichang, China by a high speed mill, and to explore the potential associations between phosphorus solubility and the structural characteristics of this phosphorite after mechanochemical activation in a shorter time.

## Materials and methods

The phosphorite used in this research is characterized by the following chemical composition (in wt.%): 22.38% P_2_O_5_^total^ (all the forms of P_2_O_5_ content of phosphorite [10]), 3.9% P_2_O_5_^ass^ (assimilable phosphorus content in 2% citric acid solution [10]), 2.28% F, 44.67% CaO, 2.89% R_2_O_3_ (R = Al, Fe), 0.75% Na_2_O, 0.063% SO_3_, 7.51% SiO_2_, 2.54% MgO, 7.56% CO_2_, moisture content of 1.25% and average granulometric particle size of 0.15 mm. There are three forms of P_2_O_5:_ water-soluble, assimilable, and insoluble in water. The assimilable forms of P_2_O_5_^ass^, which is an indicator for the successful application of a phosphorus fertilizer, is insoluble in water, but soluble in the soil solutions, which can be assimilated by plants. Phosphorus solubility in citric acid was determined by the following Eq (1):

$$\mathrm{Phosphorus}\;\mathrm{solubility}\;(\%)=\frac{P_{2}O_{5}{}^{ass}}{P_{2}O_{5}{}^{total}}\times 100\%$$ (1)

The mechanochemical activation of phosphorite in a planetary mill (produced in Transformation Center of China and Russia in Jiaxing Zhejiang Province), with two 242 cm^3^ chambers, was performed with a milling speed of 1,500 revolutions per minute (rpm). Each chamber was filled with a sample of 5 g weight and five steel balls with a diameter of 18 mm that totally weighed 150g. The milling times were set to 0, 5, 10, 15, 20, 25, 30, 35, 40, 45, 50, 55 and 60 min.

The surface structural measurements were conducted by scanning electron microscope (SEM) on a JSM-7000F (JEOL, Japan) with an accelerating voltage of 0–30000 Volt and a resolution of 1–3 nm. All samples were coated with gold by sputtering prior to observation.

The infrared spectroscopy (IR) was performed in the spectral range of 450–4000 cm^-1^ with Perkin-Elmer-FT-1730 using KBr pellets. A resolution of 4 cm^-1^ was used collecting 16 scans for each sample.

The powder XRD data were collected with the Bruker D8 ADVANCE diffractometer (2θ 20°–60°, λ = 0.15046Å, step 0.02°, count time 0.5s/step, Cu Kα radiation). The peak intensity, half peak width (β), and d-spacing values of the peaks in the XRD patterns of the samples were determined using the MDI Jade 5.0 program. The mean crystallite size (D) was calculated from XRD with the Scherrer Eq (2) [11].

$$D=\frac{\lambda }{\beta \cos\theta }$$ (2)

D (nm), mean crystallite size

λ (nm), wavelength, 0.15046

β (°), half peak width

θ (°), the diffraction angle

The microstrain (ε) calculated by the Bragg Eq (3) was correlated with the half peak width (β) [11].

β=εtgθ (3)

β (°), half peak width

θ (°), the diffraction angle

ε, microstrain

The unit-cell parameters (a, b, c) were refined using the program MDI Jade 5.0. The PDF (Powder Diffraction File, ICDD, 2004) database was used for determination of the phases and minerals present in the samples.

The powder XRD data were analyzed by MDI Jade 5.0 programs and the figures were plotted by OriginLab 8.6 program.

## Results

### Phosphorus solubility in citric acid determination

**Fig 1:**
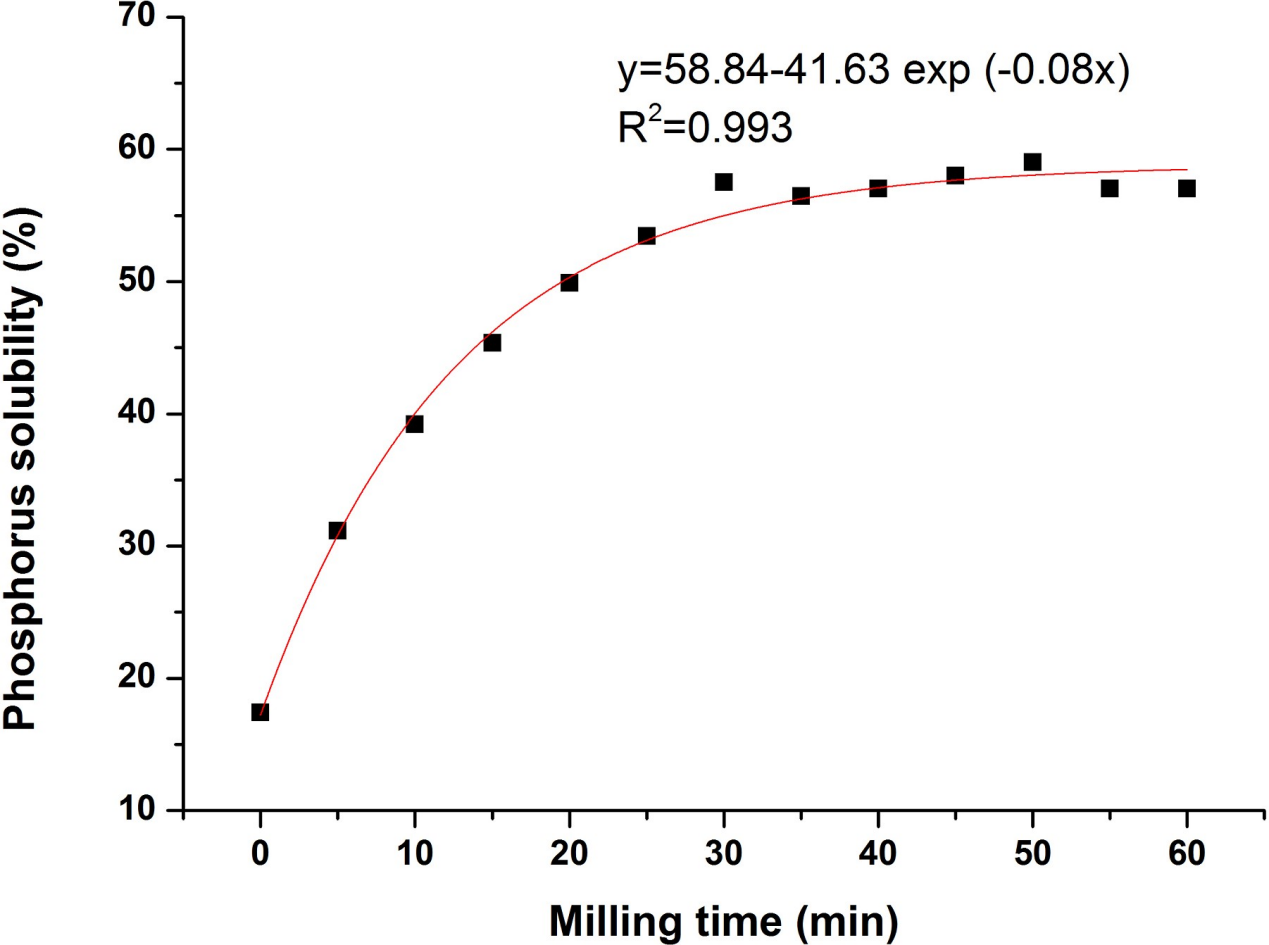
Significant dependence of the solubility of phosphorus originating from phosphorite in citric acid on the milling time from 0 to 60 min.

Besides the total phosphorus content (P_2_O_5_^total^), phosphorus solubility in citric acid is one of the most important factors to evaluate whether the phosphorite can be used as fertilizers. Compared with the initial phosphorite solubility (17.43%), phosphorus solubility in citric acid varied from 31.14% to 59.03% as a result of milling time between 5 and 60 min (Fig 1). Phosphorus solubility in citric acid grew significantly with the milling time from 0 to 60min (Fig 1). A rapid increase in phosphorus solubility during the first 30 min of milling was observed, and it has almost reached a maximum (59.03%) in 30 min (57.51%). There were no distinct changes in phosphorus solubility from 30 to 60 min.

### Surface morphology observation

**Fig 2:**
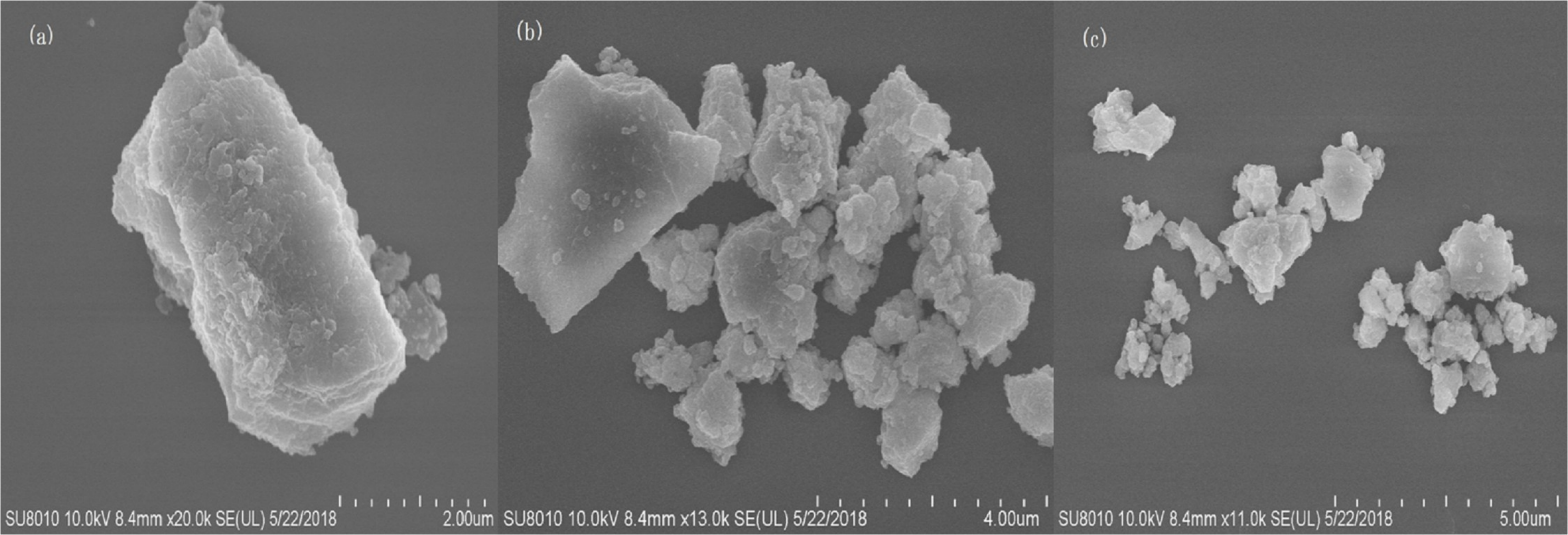
SEM image of Yichang phosphorite samples: (a) inactivated, marker 2μm; (b) 30 min activated, marker 4μm; (c) 60 min activated, marker 5μm.

The analysis with scanning electron microscopy (SEM) was made with an intent of providing the information about the changes in the dispersion and surface morphology of the samples (Fig 2). Compared with the phosphorite before mechanochemical activation the phosphorite that was milled for 30 and 60 min showed different changes in the surface morphology of the samples as assessed by SEM. The shape of the phosphate ore before milling was characterized by rough surface, compact structure, high degree of crystallinity, and a greater number of edges and corners (Fig 2A), while the apatite had a good spherical shape and looser structure, and the surface boundaries of the grains became blurred after mechanochemical activation (Fig 2B and 2C). The SEM image of the phosphorite activated for 30 min showed that the phosphorite tended to form isometric particles, and the minimum particle size decreased to about 100 nm. With the increase of milling time up to 60 min, more spherical particles were clearly visible and the particle size no longer decreased.

### Infrared spectroscopy analysis

**Fig 3:**
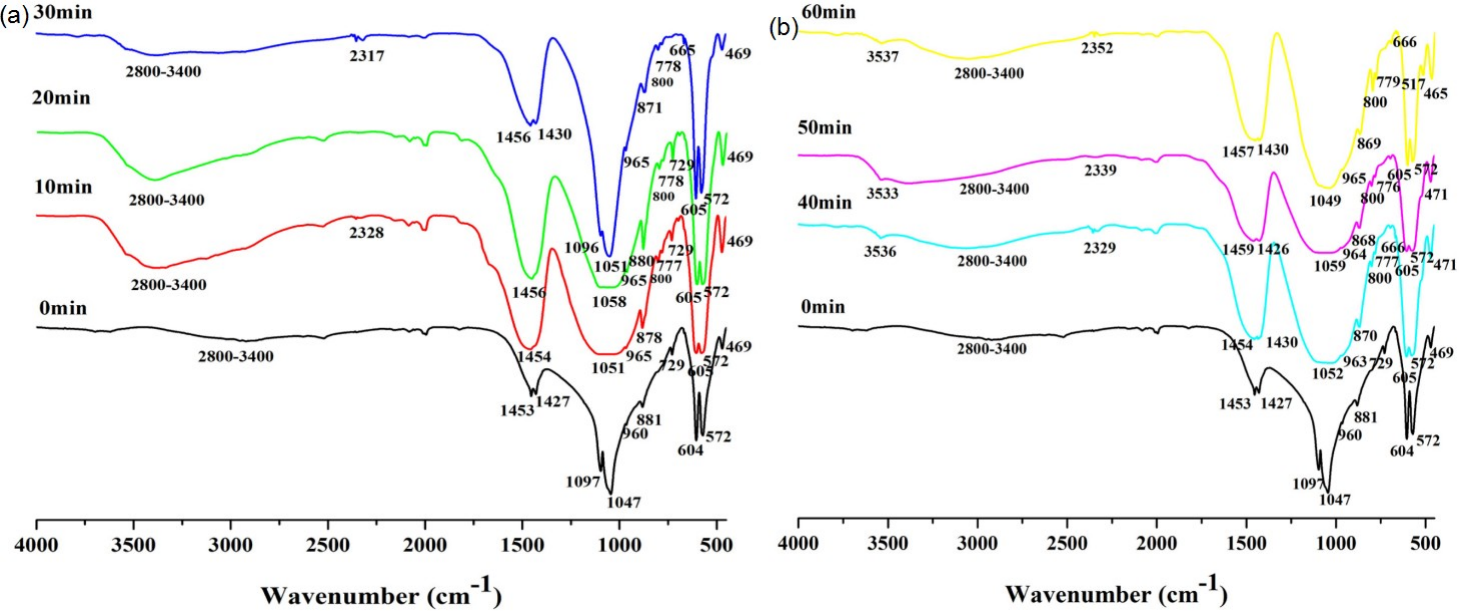
IR spectra of apatite with different milling time: (a) activated for 0, 10, 20, 30 min and (b) activated for 0, 40, 50, 60 min.

The infrared spectroscopy (IR) analysis (Fig 3 and Table 1) detected functional groups such as OH^-^, CO_3_^2-^, and PO_4_^3-^ ions as well as new peaks in the natural phosphorite for different activation times. In the spectra of the non-activated phosphorite, the existence of PO_4_^3-^ ions was detected in the absorption band of 960 cm^-1^ (ν1) and in the two splitting absorption bands of 572-604cm^-1^ (ν4) and 1050–1100 cm^-1^ (ν3). The absorption bands of carbonate ions were also detected (ν2 = 860–880 cm^-1^, ν3 = 1400–1470 cm^-1^ and ν3 = 729 cm^-1^). The absorption bands were displayed with regard to the presence of H_2_O and SiO_2_ (2800–3400 cm^-1^ and 470 cm^-1^, respectively). With the extension of activation time, the absorption bands of functional groups in the samples changed significantly. These spectra reflected the lowering of the absorption bands intensity (ν4 = 572–604 cm^-1^ and ν1 = 960cm^-1^) belonging to the stretching vibrations of PO_4_^3-^ ions with the increase of milling time. The absorption bands of stretching P-O-P (ν3) at 1050 cm^-1^ and asymmetric bending O-P-O (ν4) at 517 cm^-1^ confirmed that the new phase of β-Ca(PO_3_)_2_ generated with the prolonged milling time. The spectra showed that the intensity of the absorption bands belonging to stretching vibration of CO_3_^2-^ (ν3 = 1400–1470 cm^-1^) decreased during the activation. The intensity of the absorption bands of CO_3_^2^ at 729 cm^-1^ demonstrated an increasing trend for the first 20 min, but disappeared after milling for 30 min. The new OH^-^ ion absorption bands (3530 and 660 cm^-1^) were detected in the apatite structure activated for more than 30 min, which indicated an emergence of fluorine-hydroxide bonds(-F…HO-). The absorption bands at about 770–800 cm^-1^ and 2300 cm^-1^ belonging to Si-O-Si (ν1) and O-C-O (ν3) was visible once the samples were milled.

**Table 1:** The main characteristics of IR absorption bands of apatite with different milling time.

No.	Band position (cm-1)							Vibrational mode
	0min	10min	20min	30min	40min	50min	60min	
**1**	469	469	469	469	471	471	465	Symmetric O–Si–O (ν2) bending mode in α-SiO_2_
**2**	-	-	-	-	-	-	517	Asymmetric O–P–O (ν4) bending mode in β-Ca(PO_3_)2(60 min)
**3**	572	572	572	572	572	572	575	Doubly degenerate asymmetric O–P–O (ν4) stretching mode in CFAp
	604	605	605	605	605	605	602	
**4**	-	-	-	665	666	-	666	OH libration mode of OHFAp (and/or CFOHAp) (30, 40, 60 min)
**5**	729	729	729	-	-	-	-	O–C–O (ν4) bending mode in CaCO_3_ and B-type CO_3_^2-^ in CFAp
**6**	-	777	778	778	777	776	779	Degenerate symmetric Si–O–Si (ν1) stretching mode in α-SiO_2_ (10–60 min)
	-	800	800	800	800	800	800	
**7**	881	878	880	871	870	868	869	Symmetric O–C–O (ν2) bending mode in CaCO_3_ and B-type CO_3_^2-^ in CFAp
**8**	960	965	965	965	963	964	965	Symmetric P–O–P (ν1) stretching mode of PO_4_^3-^ in CFAp (and/or CFOHAp)
**9**	1047	1051	1058	1051	1052	1059	1049	Asymmetric P–O–P (ν3) stretching mode of PO_4_^3-^ in CFAp (0,30min)(and/or CFOHAp), of PO_3_^2-^ in β-Ca(PO_3_)_2_ (10, 20, 40, 50, 60 min)
	1097	-	-	1096	-	-	-	
**10**	1427	-	-	1430	1430	1426	1430	Doubly degenerate asymmetric O–C–O (ν3) stretching mode of B-type CO_3_^2-^ in CFAp (and/or CFOHAp) and/or CaCO_3_
	1453	1454	1456	1456	1454	1459	1457	
**11**	-	2328	-	2317	2329	2339	2352	Degenerate asymmetric O–C–O (ν3) stretching mode in CO_2_ (air)
**12**	2800–3400	2800–3400	2800–3400	2800–3400	2800–3400	2800–3400	2800–3400	Symmetric OH^-^(ν1) stretching mode in crystal water
**13**	-	-	-	-	3536	3533	3537	Symmetric OH^-^ (ν1) stretching mode in structure associate water

### Powder XRD phase and structural analysis

**Fig 4:**
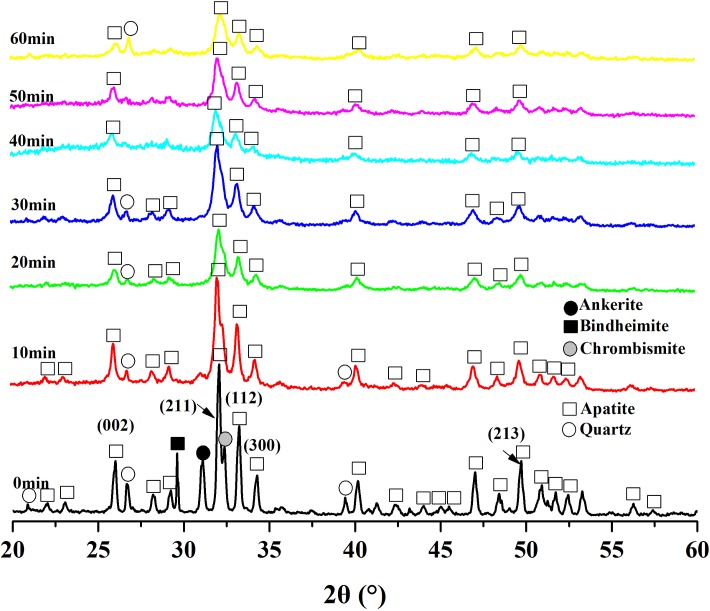
XRD patterns of phosphorite at different milling time.

The XRD diffraction patterns of the ground phosphate rocks changed with the different milling time (Fig 4). The pattern analysis of the initial phosphorite, confirmed that there was a major phase, apatite and impurities of quartz, calcite, ankerite, and chrombismite. The relative intensities of all the associated mineral reflections decreased remarkably with the increased milling time, and some even disappeared except for the quartz. The intensities of the apatite reflections were also decreased and the widths of the peaks increased. Both the apatite and accompanying minerals became amorphous with the prolonged milling time.

**Fig 5:**
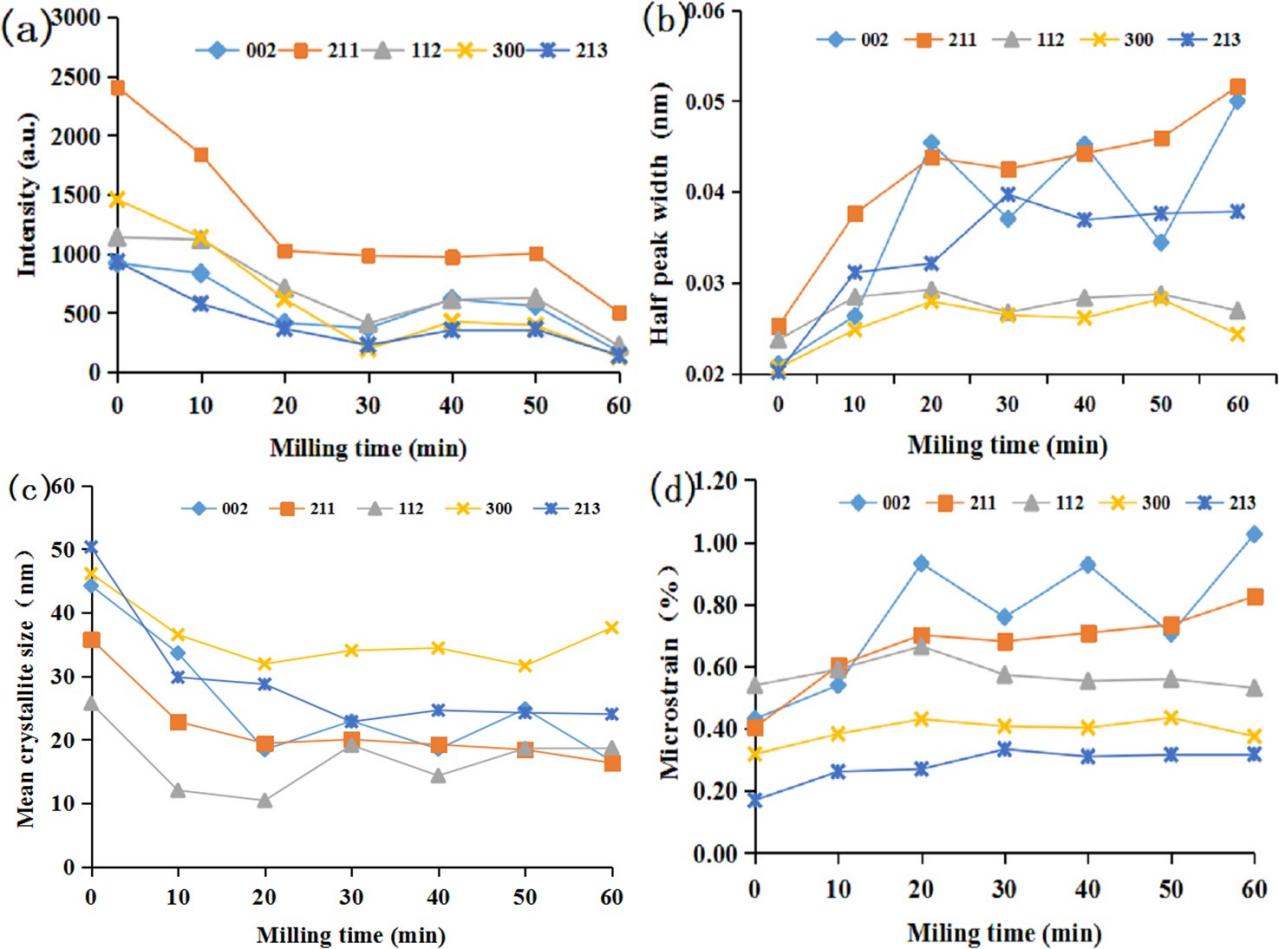
Changes in the main apatite crystal surfaces depending on the milling time detected by XRD: (a) peak intensity; (b) half peak width; (c) mean crystallite size; (d) microstrain.

During the activation for the first 20 min, the peak intensity and crystallite size of the main lattice faces dropped rapidly, and even disappeared after milling for 60 min. Then, they slowly decreased in the subsequent 40 min of milling with no decrease of crystallite size (Fig 5). On the contrary, both the half peak width and the microstrain had a certain degree of increase as the function of milling time. They increased rapidly in the first 20 min and decreased gradually after 20 min (Fig 5).

**Fig 6:**
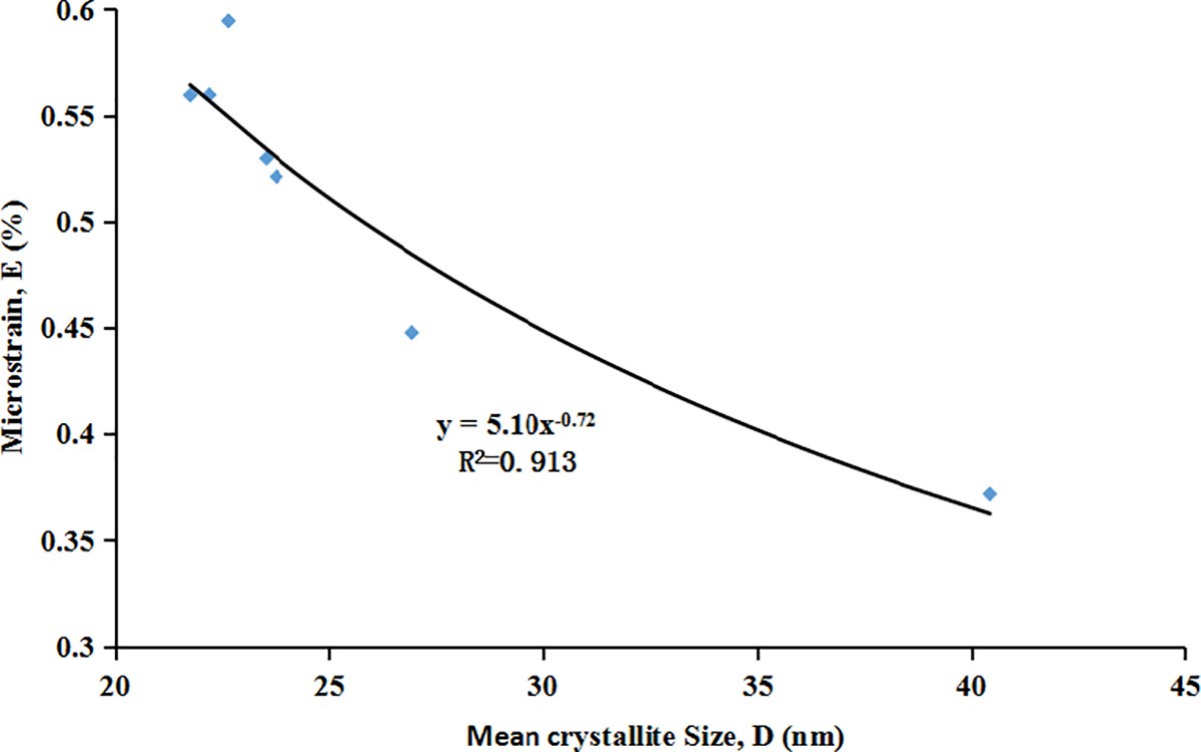
Correlation between mean crystallite size, (D) and microstrain, (E) in apatite.

It is evident that there is an inverse correlation between the mean crystallite size (D) of apatite caused by milling and the microstrain (E) (Fig 6). That is smaller the crystallite size, greater the lattice distortion. The mathematical correlation between D and E was calculated with the following equation:

$$E=5.10\;D^{-0.72}$$

The mineral composition including apatite and impurity in the samples with different milling time were determined according to d-spacing values and XRD phase analyses (Tables 2 and 3). These data verified the presence of a major phase, fluorapatite (FAp) and the minor content small amount of quartz and CaCO_3_ in the initial sample. New phases such as hydroxyapatite (Ca_5_(PO_4_)_3_OH, OHAp) and carbonate-apatite (Ca_10_(PO_4_)_6_CO_3_, CAp) were formed with increase in the activation time for more than 20 min. The ratios of the peaks intensities of quartz and calcite to the peaks intensity of apatite (Ap) in XRD patterns reflected the changes in impurity content in the powders. The calcite to apatite ratio remarkably dropped even by 10-fold during the course of mechanochemical activation, while the quartz to apatite ratio o considerably decreased in the first 50 min but slightly increased after milling for 60 min.

**Table 2:** The d-spacing values and XRD phase analyses of the major and minor phases of phosphorite.

Duration of MA (min)	Identified phases (nm)
0	Ca_5_F(PO_4_)_3_ (15–0876) (FAp): 0.344, 0.280*, 0.270, 0.262 α-Quartz (46–1045): 0.334*, 0.228, 0.425 CaCO_3_ (47–1743): 0.303*, 0.193, 0.187
10	Ca_5_F(PO_4_)_3_ (15–0876) (FAp): 0.344, 0.280*, 0.270, 0.262 α-Quartz (46–1045): 0.334*, 0.228, 0.212 CaCO_3_ (47–1743: 0.303*, 0.193, 0.187
20	Ca_5_F(PO4)_3_ (15–0876) (FAp): 0.344, 0.277, 0.270*, 0.262 α-Quartz (46–1045): 0.334*, 0.228, 0.212 CaCO_3_ (47–1743: 0.303*, 0.193, 0.187 Ca_5_(PO_4_)_3_OH (09–0432) (OHAp): 0.278*,0.344, 0.226 Ca_10_(PO_4_)_6_CO_3_ (35–0180) (CAp): 0.381, 0.278*, 0.274
30	Ca_5_F(PO_4_)_3_ (15–0876) (FAp): 0.344, 0.277, 0.280*, 0.270 α-Quartz (46–1045): 0.334*, 0.228, 0.212 CaCO_3_ (47–1743: 0.303*, 0.193, 0.187 Ca_5_(PO_4_)_3_OH (09–0432) (OHAp): 0.278*,0.344, 0.184, 0.194 Ca_10_(PO_4_)_6_CO_3_ (35–0180) (CAp): 0.381, 0.278*, 0.343
40	Ca_5_F(PO_4_)_3_ (15–0876) (FAp): 0.344, 0.277, 0.280*, 0.270 α-Quartz (46–1045): 0.334*, 0.197, 0.212 CaCO_3_ (47–1743: 0.304*, 0.187, 0.228 Ca_5_(PO_4_)_3_OH (09–0432) (OHAp):, 0.278*,0.344, 0.270, 0.281 Ca_10_(PO_4_)_6_CO_3_ (35–0180) (CAp): 0.381, 0.278*, 0.343,0.246
50	Ca_5_F(PO_4_)_3_ (15–0876) (FAp): 0.344, 0.277, 0.280*, 0.270 α-Quartz (46–1045): 0.228*, 0.197, 0.212 CaCO_3_ (47–1743): 0.187*, 0.160, 0.193 Ca_5_(PO_4_)_3_OH (09–0432) (OHAp):, 0.278*,0.344, 0.184, 0.194 Ca_10_(PO_4_)_6_CO_3_ (35–0180) (CAp): 0.278*,0.275, 0.343,0.229
60	Ca_5_F(PO_4_)_3_ (15–0876) (FAp): 0.183, 0.277, 0.2624, 0.270* α-Quartz (46–1045): 0.334*, 0.245, 0.181 CaCO_3_ (47–1743): 0.2495, 0.303*, 0.187 Ca_5_(PO_4_)_3_OH (09–0432) (OHAp): 0.278*,0.194, 0.172,0.178 Ca_10_(PO_4_)_6_CO_3_ (35–0180) (CAp): 0.274, 0.278*, 0.343,0.3805

* the d-spacing values of the strongest peaks of different minerals.

FAp, fluorapatite. OHAp, hydroxyapatite. CAp, carbonate-apatite.

**Table 3:** Ratios of the peaks intensities of quartz and calcite to the peaks intensity of apatite in XRD patterns.

Milling time (min)	Quartz/Ap	Calcite/Ap
0	0.69	0.57
10	0.05	0.06
20	0.27	0.05
30	0.27	0.06
40	0.37	0.12
50	0.30	0.06
60	0.75	0.14

Ap, apatite.

Data analysis performed for determination of the unit-cell parameters of apatite are given in Table 4. The calculated unit-cell parameters of the initial flourapatite component were: a = 9.343 Å and c = 6.869 Å. The mechanochemical activation caused a decrease in the unit cell parameters “a” and “c” of the apatite, for -0.01 mean, value (except for a slight increase by +0.004 for the 40 min and +0.005 for the 30 min activation).

**Table 4:** Unit-cell parameters (a, b, c) of apatite present in the studied phosphorite subjected to mechanochemical treatment of various duration.

Milling time (min)	0	10	20	30	40	50	60
**a (Å)**	9.343	9.333	9.325	9.334	9.347	9.339	9.337
**b (Å)**	9.343	9.333	9.325	9.334	9.347	9.339	9.337
**c (Å)**	6.869	6.865	6.835	6.874	6.859	6.867	6.858

## Discussion

Our results showed that the solubility of phosphorus from apatite ores in citric acid increased proportionately with the grinding time, and it nearly reached the maximum level (59.03%, 50 min) at 30 min (57.51%) and then gradually reached an equilibrium, under the condition of the constant other processing parameters. In our previous study, the maximum of phosphorus solubility in citric acid of Huangmailing phosphorite was 46.53% after milling for 21 min by a planetary ball mill [9]. Petkova et al. (2015) used a an exponential equation to describe the relationships between the phosphorus solubility, which reached the maximum (60%) at 300 min and milling time [7]. According to the fertilizer criterion in Brazil [12], phosphorite can be applied in the field when the phosphorus solubility in citric acid is more than 30%. In our study, the phosphorus solubility reached more than 31.14% after milling for 5 min, which exceeds Brazilian standards. In this study, phosphorite milling for more than 30 min, can give results equivalent to calcium superphosphate, considering the amounts of assimilable phosphorus (P_2_O_5_>12%). Considering the amount of power consumption and the effectiveness of phosphorite processing, milling for 30 min was found to be most feasible in this experiment. Unlike in the previous studies (where milling time was more than 150 min) [5, 8], our research with a milling time of 30 min was more efficient and promising. The next step in such research should include the verification of the bioavailability of mechanochemically activated phosphorite in field conditions.

Changes in surface morphology after mechanochemical activation of phosphorite recorded by SEM, including particle size reduction to about 100 nm, loose structure and fuzzy surface boundary, were similar to the results of Yaneva et al. [5, 9].

The IR spectra reflected the changes in functional groups which determined the chemical composition of the studied samples and the transformation of phosphorite structure with the different activation time. In the initial sample, absorption bands of 729 cm^-1^, 881 cm^-1^ and 1425–1459 cm^-1^ indicated that the CO_3_^2-^ ions existed in the structure of calcite, dolomite and apatite [5, 10,13]. This is in accordance with the results of phase analysis obtained by XRD. The absorption bands of 2800–3400 cm^-1^ in the sample was belonged to the adsorbed water [14]. According to the number, position, strength and shape of the absorption bands belonging to the PO_4_^3-^, CO_3_^2-^, and OH^-^ ions, the initial phosphorite (with high CO_2_ content) can be defined as a carbonate fluorine (CFAp) with some amounts of adsorbed water because of its sedimentary origin [6]. With the prolonged milling time, the intensity of the absorption bands belonging to the CO_3_^2-^ ions (869-881cm^-1^) decreased and, even disappeared (729 cm^-1^). It was indicated that the crystal structures of the associated minerals, such as calcite (CaCO_3_) and dolomite (CaMg(CO_3_)_2_) were destroyed by mechanochemical activation. This was identified with the results from XRD. It was suspected that the decreasing and destroying of calcite and dolomite crystals might reduce the consumption of citric acid, and that there was enough citric acid to dissolve the apatite to increase the phosphorus solubility phosphorus in citric acid. The absorption peaks belonging to the CO_3_^2-^ ions (1425–1459 cm^-1^) of the apatite were wider and relocated, which is a characteristic of PO_4_^3-^ substitution by CO_3_^2-^ (Type B) and for the incorporation of CO_3_^2-^ resulted from the destroyed calcite and dolomite crystals, not only CO_2_ from the air (2300 cm^-1^) during the mechanochemical activation [7]. The level of substitution of CO_3_^2-^with PO_4_^3-^ should be studied in future. With the extension of the activation time for more than 30 min, the absorption bands of the newly generated OH^-^ ions (3530 and 660 cm^-1^) in the apatite structure, which indicated the formation of the fluorine-hydroxide bonds (-F…HO-), were perhaps detected due to adsorbed water in apatite structure, not only the absorption of water vapor from the air [7]. It can be concluded that the hydroxyapatite formed partly during the mechanochemical activation which was consistent with our previous results before [9]. The generation of new absorption bands of 517cm^-1^ was an evidence of the formation of a new phase β-Ca(PO_3_)_2_ in the sample with milling for 60 min. It was consistent with the results of Yaneva et al, milling for 30 min and 150 min [5]. The increase in the absorption at about 800 cm^-1^ belonging to Si-O-Si (ν1) was visible with longer milling time. Steady properties of quartz during the milling may be due to its hardness (Mohs’scale of hardness, MSH = 7) [6].

XRD diffraction patterns showed the immediate changes in the structural characteristics such as crystallite size, microstrain, unit-cell parameters and phase transformation of apatite and the associated minerals during the mechanochemical activation. The results of the phase analysis of XRD diffraction patterns showed the existence of a major phase, in apatite and accompanying minerals including quartz, calcite, ankerite and chrombismite. We proved this with the results of IR analysis. All the minerals including apatite and associated minerals became more amorphous, and even disappeared with prolonged milling time. Our previous XRD results showed that increase in half peak width and decrease in diffraction intensity and crystallite size of apatite after mechanical activation was evident [9]. It was observed that the phosphorus solubility of ores was inversely proportional to peak intensity and mean crystallite size, and directly proportional to half peak width and microstrain. The crystallite size of apatite was inversely proportional to the variation of microstrain, which further revealed the mechanism of mean crystallite size and lattice distortion. This means that the smaller the mean crystallite size is, the greater the microstrain is [15]. It was also an intrinsic mechanism that improved phosphorus solubility in citric acid. A new phase of Ca_5_(PO_4_)_3_OH (or/and Ca_10_(PO_4_)_5_CO_3_OHF) was generated with the activation by XRD, which was consistent with the results of IR. Concluding the results of powder XRD and IR spectroscopy, it is possible that the non-activated carbonate-fluorapatite (type B) was transformed into a mixture of carbonate-fluorapatite, hydroxyapatite, fluorocarbon hydroxyapatite and/or carbonate apatite respectively. However, accurately finding out the isomorphous substitution type during the mechanochemical activation of phosphorite requires additional research. It was essential that both “a” and “c” constants of the initial phosphorite were lower than the reported values for fluorapatites, which for “a” was around 9.37 Å and for “c” around 6.88 Å [6,16,17]. Both of the unit-cell parameters “a” and “c” of the phosphorite decreased after mechanochemical activation (except “a” in the 40min, “c” in the 30 min treatment), which already had a higher “c” value before milling. All the variation in the parameters “a” and “c” were attributed to deformations in the apatite crystal structure after the mechanical activation [3]. It has been concluded that the apatite cell parameter “a” gradually decreases with the increase of the CO_3_^2-^ entering the lattice because of the isomorphism, which means that the size of apatite crystallite gradually reduces [6].The more carbonate ions enter the apatite structure, the more deformed the apatite crystallite becomes. It has been documented that the substitution of phosphate by type B carbonate leads to a decrease in the unit-cell parameter “a” and increase in “c” parameter, whereas the substitution of the channel F^-^ (or OH^-^) by type A carbonate results in increase in “a” and decrease in “c” parameter [6,18,19].

Also, there was a significant dependence of phosphorus solubility of phosphorite on the structural changes. According to the results of IR and XRD, the main phase apatite and the associated minerals such as calcite, dolomite and quarts became to be amorphous during the mechanochemical activation. That means that the integrity and symmetry of apatite crystals were destroyed, indicating that the phosphorus solubility in citric acid of the samples increased after the activation. During the mechanochemical activation, PO_4_^3-^ was substituted by CO_3_^2-^ (Type B) for the incorporation of CO_3_^2-^. Because of the different radius of CO_3_^2-^ and PO_4_^3-^ ions, the isomorphous rearrangement process resulted in lattice defects of apatite, reduced particle size, and increased phosphorus solubility [8]. The more carbonate ions enter the apatite structure, the more deformed the apatite crystallite becomes. For the lower hardness of calcite and dolomite, it was suspected that the depletion of calcite and dolomite might reduce the consumption of citric acid and that there was enough citric acid to dissolve the apatite to increase the phosphorus solubility. This is why the solubility of phosphorus from apatite ores in citric acid increased proportionately during the first 30 min of milling time. With the extension of activation for more than 30 min, the hydroxyapatite was partly formed after the addition of OH^-^. The addition of OH^-^ ions into the apatite structure caused it to be easier to destruct along the planes of (2 1 1), (3 0 0), (2 0 2) [6]. The defects of apatite for the incorporation of CO_3_^2-^ and OH^-^ caused the increasing of phosphorus solubility of phosphorite. The solubility of phosphorus from apatite ores in citric acid gradually reached an equilibrium after milling for 30 min, which was also consistent with the changes of particle size, called milling limit [13] during the mechanochemical activation.

In addition, during mechanochemical activation, the impact force, shear force, and other factors, could lead to the plastic deformation and reduced crystallinity of apatite, including dislocation, deformation, recrystallization, defects and even formation of amorphous substances [13]. The reason for this may also be the high local temperature and pressure between milling media and the ore particles in a high-energy and unstable state, during the process of mechanical activation. Also, a large amount of mechanical energy is loaded into the crystal lattice, which causes the chemical reaction between the particles [20]. According to Criado et al. (1986) [11], the chemical reactivity of solids is strongly dependent on both the mean crystallite size and the concentration of microstrains, which can be modified by mechanochemical treatments of the samples.

## Conclusions

The chemical analysis reveal that the effect of mechanochemical activation of Yichang phosphorite in a planetary mill depends on the milling time with the fixed other processing parameters. With the first duration of 30 min, phosphorus solubility in citric acid increased significantly, then grew slowly and tended to be balance in the next 30 minutes.

In coordinating to the effectiveness and power consumption of mechanochemical activation in our study, the effective milling time (30 min) was chosen.

The changes in surface morphology, surface groups, crystal structure and phase composition of phosphorite are in correlation with the phosphorus solubility in citric acid, during the mechanochemical activation. This is mainly demonstrated through considerably reduced particle size, decreased crystallinity of the apatite and increased structural defects due to substitution of PO_4_^3-^ with CO_3_^2-^ and the incorporation of OH^-^.
